# Epigenetic Legacy: The Role of Sperm miRNAs in the Paternal Inheritance of Diabetes and Obesity Development

**DOI:** 10.1002/dmrr.70157

**Published:** 2026-03-23

**Authors:** Katharina Laurent, Raffaele Teperino, Martin Hrabě de Angelis, David A. Skerrett‐Byrne, Johannes Beckers

**Affiliations:** ^1^ Helmholtz Zentrum München, German Research Center for Environmental Health Institute of Experimental Genetics Neuherberg Germany; ^2^ German Center for Diabetes Research (DZD) Neuherberg Germany; ^3^ Helmholtz Zentrum München, German Research Center for Environmental Health (GmbH) German Mouse Clinic Neuherberg Germany; ^4^ Chair of Experimental Genetics, TUM School of Life Sciences Technische Universität München Freising Germany; ^5^ Reproductive and Family Health Program Hunter Medical Research Institute Awabakal Country, Newcastle New South Wales Australia; ^6^ School of Biomedical Sciences and Pharmacy, College of Health, Medicine and Wellbeing The University of Newcastle Awabakal Country, Callaghan New South Wales Australia; ^7^ INFRAFRONTIER ERIC Neuherberg Germany

**Keywords:** diabetes, epididymis, epigenetics, miRNAs, obesity, shiny app, sperm

## Abstract

In recent decades, obesity and diabetes have reached pandemic levels, with obesity now recognised as a major health risk factor. Evidence shows that metabolic diseases are more pronounced in the offspring of malnourished parents, suggesting that predisposition can be inherited via epigenetic information in gametes. This has sparked growing interest in small regulatory RNAs in sperm as carriers of epigenetic inheritance. However, the functional annotation of dysregulated sperm microRNAs (miRNAs) in obesity and diabetes remains limited. This work addresses this gap by analysing publicly available datasets of diet‐regulated sperm miRNAs and linking them to genes functionally associated with obesity and diabetes. We systematically identified diet‐responsive sperm miRNAs and overlapped their predicted targets with genes associated with metabolic phenotypes, as catalogued by the International Mouse Phenotyping Consortium (IMPC). First, in a sequence‐based approach, we uncovered 11,272 and 6528 potential target genes for miRNAs regulated by the acute and chronic HFD interventions, respectively. Second, by overlapping these predicted target genes of sperm miRNAs with our IMPC‐derived list of 889 genes associated with obesity and diabetes, we identified 805 acute‐ and 546 chronic‐HFD predicted response genes. This approach thus associates function with regulated miRNAs and revealed distinct miRNA–gene networks in acute versus chronic HFD models, including shared nodes in pathways related to insulin signalling, lipid metabolism, and β‐cell function. To support further research, we provide the field with the *ShinyFatSperm* App (https://reproproteomics.shinyapps.io/ShinyFatSperm/), which facilitates the functional interpretation of diet‐regulated sperm miRNAs and enables users to explore their roles in the intergenerational transmission of metabolic disease risk. Taken together, our findings reinforce the concept that paternal dietary exposures can influence offspring health through epididymal‐ and sperm‐borne miRNAs, and related epigenetic mechanisms. This work provides a roadmap for hypothesis‐driven investigation into the intergenerational inheritance of metabolic diseases and highlights the urgent need for translational strategies to interrupt this cycle.

## 1 Introduction

### 1.1 From Epidemiology to Inheritance: The Case for Paternal Metabolic Programming

Globally, the prevalence of obesity and diabetes has reached pandemic proportions in recent decades, with obesity recognised as one of the most significant health risk factors in modern societies [1]. It is driven by a complex interplay of genetic, environmental, behavioural, and metabolic influences. Key contributors include the widespread availability of so‐called ‘Western diets’ combining high fat and sugar content, the consumption of highly processed foods, and excessive sugar intake [2]. Obesity is associated with a range of comorbidities including high blood pressure, cardiovascular disease, and type 2 diabetes mellitus (T2DM). Reflecting this, the International Diabetes Federation (IDF, https://diabetesatlas.org/) Diabetes Atlas reports that 589 million people are now living with diabetes, incurring over one trillion USD in health expenditure, a striking 338% increase over the past 17 years. In Europe alone, diabetes‐related expenditure amounts to $192.9 billion USD, representing 19.0% of the global total [3].

With the rise of obesity and T2DM, substantial efforts have been directed towards understanding their origins and key risk factors. Studies show that children of parents with obesity and T2DM have a higher lifetime risk of developing these conditions compared with those without such parental history [4]. A well‐established factor is the inheritance of genetic traits, as demonstrated by numerous GWAS [4, 5, 6, 7]. However, this alone cannot explain the pandemic development. Instead, emerging evidence shows that offspring predisposition can also be inherited via epigenetic information in gametes [4, 8]. Research on the developmental origins of health and disease (DOHaD) has so far focused predominantly on maternal contributions [9, 10], partly because maternal metabolism directly affects the embryo during pregnancy and breastfeeding. In contrast, this review aims to draw attention to the underrepresented paternal aspect, namely sperm. Notably, studies have shown that RNA microinjection from the sperm of Western diet‐fed mice into rodent zygotes can induce obesity and T2DM in the offspring [11], highlighting the critical role of paternal factors in the intergenerational transmission of metabolic disease.

**FIGURE 1:**
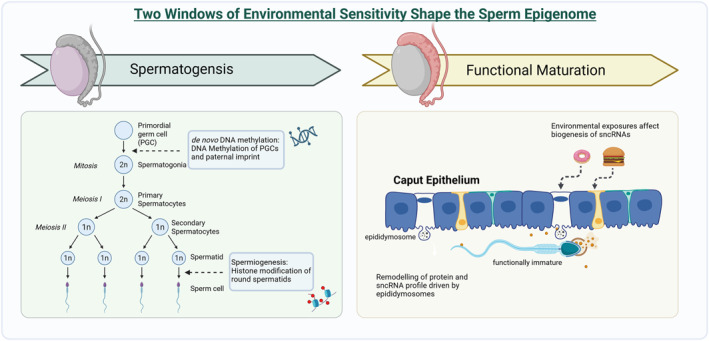
Two windows of environmental sensitivity shape the sperm epigenome. On the left is an illustrative graph of spermatogenesis, highlighting windows of sensitivity during the *de‐novo* DNA methylation and spermiogenesis. On the right is the second window of sensitivity illustrating environmental impacts on the caput epithelium during functional maturation. Aspects of this figure were created with BioRender.com.

During spermatogenesis and the functional maturation of sperm, there are two critical phases of environmental sensitivity (Figure 1). The first phase occurs during spermatogenesis in the testes, where histone modifications (e.g., methylation and acetylation) are central to paternal epigenetic programming. Disruptions in this histone landscape can have profound consequences for the next generation [12]. Once morphologically mature sperm enter the epididymis, they undergo functional maturation [13] and a second window of sensitivity begins. During this phase, sperm lose transcriptional and translational activity, and become particularly susceptible to external stimuli [13]. Functional transformation is mediated through the exchange of diverse macromolecular cargo between the spermatozoa and the surrounding luminal fluid via epididymosomes and nanotubules [14, 15, 16]. Recent studies have emphasised that the epididymis is not only essential for the maturation of sperm [14] but also for shaping its epigenetic cargo [17], which is sensitive to environmental changes [18, 19]. This window of susceptibility is localised to the proximal epididymal region, the caput [18, 20, 21], where environmental exposures induce molecular changes in the epididymal epithelium, driving the biogenesis of small non‐coding RNAs (sncRNAs) such as microRNAs (miRNAs). These RNAs are subsequently packaged and transferred to maturing sperm via epididymosomes.

miRNAs are small non‐coding RNA molecules, approximately 20–24 nucleotides in length, that play crucial roles in post‐transcriptional gene regulation. Remarkably, more than 30% of human genes are estimated to be regulated by miRNAs, underlining their broad impact on transcriptomes and proteomes [22]. miRNA expression is tightly controlled and is subject to epigenetic regulation, including DNA methylation, histone modifications, and RNA modifications [23]. This interplay creates a complex regulatory network in which miRNAs both regulate and are regulated by epigenetic mechanisms. Despite this, there is limited understanding of the downstream consequences of diet‐influenced sperm miRNAs and their potential role in epigenetic inheritance. This review aims to address this knowledge gap by investigating the associations between diet‐regulated sperm miRNAs and gene‐driven phenotypic parameters linked to obesity and diabetes.

### 1.2 The Epigenetic Legacy of Diet: How Sperm Transmits Metabolic Memory

Recent studies have highlighted the profound effects of paternal diet on offspring metabolic health, mediated through epigenetic changes in sperm small non‐coding RNAs (sncRNAs) [19, 24, 25, 26, 27, 28]. These findings provide compelling evidence that paternal nutrition can influence the metabolic trajectory of future generations. In particular, paternal overweight, often induced by chronic consumption of high‐calorie diets (Western or high‐fat diets, HFD), has been shown to exert pronounced metabolic effects in offspring [8, 11, 26, 28]. Notably, these effects persist even when offspring of HFD‐fed fathers are raised on standard diets, with obesity and insulin resistance still observed [11, 25]. This suggests that the consequences of paternal diet extend beyond the father's own health and can have lasting, heritable impacts.

One frequently used method to demonstrate this effect is microinjection of specific miRNAs into embryos, which has been shown to induce metabolic alterations [11]. However, the concentration of miRNAs used in such experiments may not accurately reflect the physiological levels found in sperm after Western diet exposure, thus highlighting the need for mechanistic insight into how environmental factors affect the sperm sncRNA profile.

The epididymis plays a critical role in this context, as it mediates the functional maturation of spermatozoa during their transit. Sperm undergo dynamic remodelling of both their proteome [14] and sncRNA content during epididymal passage [29]. Growing evidence suggests that the epididymis responds to environmental cues and transmits these signals to the maturing sperm [18, 20, 27, 30]. Although several environmental factors can influence this process, including diet, heat, stress, and toxicants such as acrylamide [18, 20, 31, 32], two key mechanisms are implicated: (1) soma‐to‐germ cell communication and (2) sperm‐borne sncRNAs.

Extracellular vesicles (EVs), known as epididymosomes, released by epididymal epithelial cells have been identified as crucial mediators. They deliver molecular signals that significantly shape the final sncRNA profile of sperm [33, 34]. In a prior study, although similar levels of DNA damage were observed, embryonic developmental failure (e.g., resorptions) occurred only when sperm had been exposed to acrylamide during epididymal transit, not during testicular spermatogenesis [18, 35]. Spermatogenesis in mice spans ∼35 days in the testis, followed by ∼14 days of epididymal maturation. RNA sequencing (RNA‐seq) of two‐cell embryos linked these developmental failures to pathway alterations, providing mechanistic insights into the consequences of epididymal exposure [18].

A follow‐up study further showed that acute acrylamide exposure significantly altered the sperm sncRNA cargo, particularly increasing miRNA content [18]. Tracing the origin of these miRNAs along the epididymis confirmed the caput (proximal) region as the main site of molecular exchange. Proteomic analyses supported this finding, identifying transcription factors likely involved in the biogenesis and transfer of miRNAs between epithelial cells, epididymosomes, and sperm [18, 20, 21].

Another study reinforced the importance of epididymal‐derived sncRNAs by showing that subchronic heat stress altered the miRNA composition of sperm, accelerating preimplantation embryonic development and modifying placental morphology, particularly in the labyrinth zone, the main site of maternal–foetal nutrient exchange [36]. Further mechanistic insights came from a corticosterone challenge model simulating prolonged stress, which revealed a complex phosphoproteomic response mediated by glucocorticoid receptors [20]. These receptors, also implicated in the acrylamide model [18], appear to orchestrate oxidative stress responses and DNA damage repair, potentially contributing to altered miRNA profiles.

The second key mechanism involves sperm‐borne sncRNAs themselves. Although sperm become transcriptionally and translationally quiescent after testicular development, mitochondrial transcription remains active, particularly for mitochondrial tRNA (mt‐tRNA) fragments. These fragments and their epigenetic consequences are receiving growing attention [19]. One recent study [19], in line with previous work [18, 35], demonstrated that HFD‐induced epigenetic effects in sperm are linked more strongly to epididymal rather than testicular development. A marked shift in the sncRNA profile of cauda spermatozoa was attributed to diet‐induced mitochondrial dysfunction, which led to increased production of mt‐tRNAs and their 5′ fragments. These changes appear to be a compensatory response and are associated with glucose intolerance in male offspring [19].

Importantly, these epigenetic modifications are not necessarily fixed. The previously described study showed that a four‐week dietary intervention following acute HFD exposure reversed many adverse effects, with offspring displaying only partial glucose intolerance [19] This suggests that diet‐induced epigenetic changes can be modifiable. Human studies support this, with one trial showing that just 1 week of a sugar‐rich diet altered the sperm RNA profile and correlated with changes in motility and obesity risk [27].

Together, these findings underscore that environmental exposures, particularly diet, can significantly influence the sperm epigenome via epididymal processes. The mechanisms are beginning to be unravelled, and they support the critical role of paternal diet in shaping offspring metabolic health through sncRNA‐mediated inheritance. To support further investigations, we compiled studies employing acute or chronic HFD feeding combined with sncRNA sequencing, listing all miRNAs altered by diet. These datasets may help interpret the phenotypic outcomes potentially driven by specific sperm miRNAs.

However, it is important to note that the majority of the previously summarised studies have been conducted in animal models, predominantly in rodents. Therefore, well‐controlled human studies are required to validate the translatability of these findings and to determine the extent to which diet‐induced alterations in the sperm epigenome contribute to offspring health in humans.

## 2 Methods

### 2.1 Literature Search

**FIGURE 2:**
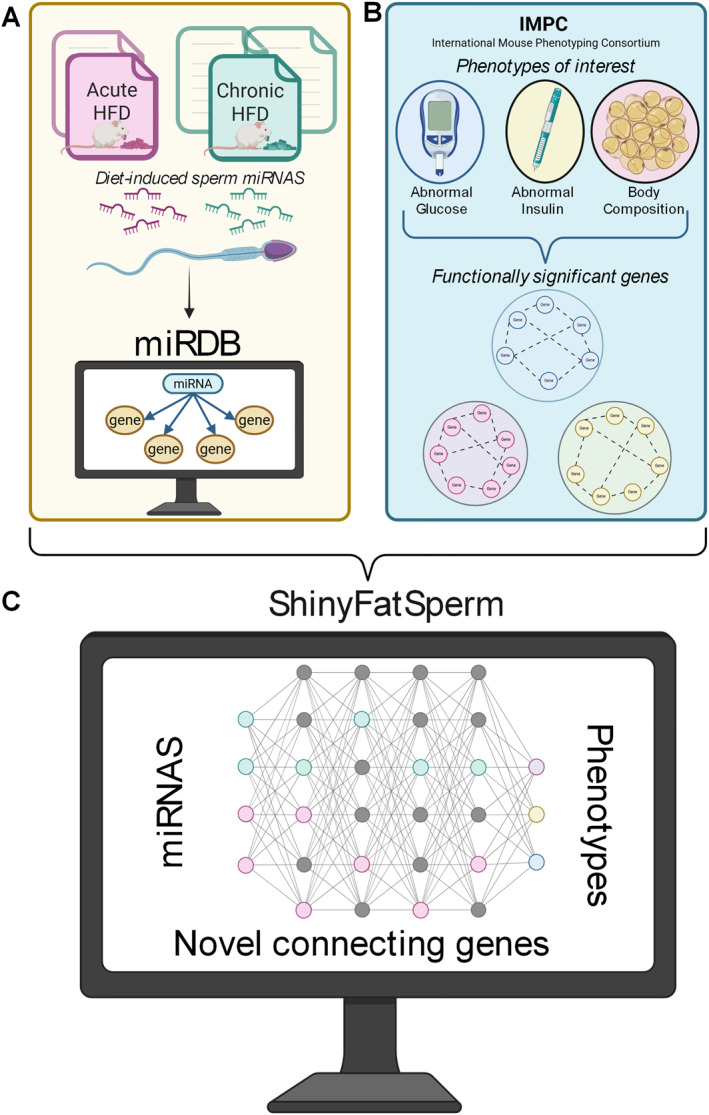
Workflow figure. (A) Literature research to identify miRNAs influenced by dietary intervention and insert them into miRDB to receive target genes. (B) Using IMPC database for collecting genes associated with phenotypes of interest. (C) Overlap of IMPC and literature data in the shiny app to identify novel connecting genes. Aspects of this figure were created with BioRender.com.

Academic searches were conducted in PubMed using the following keyword combination: (‘mice’ OR ‘mouse’ OR ‘Mus musculus’) AND (‘HFD’ OR ‘high‐fat diet’ OR ‘Western diet’ OR ‘obesity’ OR ‘diabetes’ OR ‘diet‐induced obesity’) AND (‘intergenerational’ OR ‘epigenetic inheritance’ OR ‘sperm’ OR ‘miRNAs’) (Figure 2A). The search was restricted to publications in English.

We included studies based on chronic or acute high‐fat diet (HFD) feeding regimens that provided data on sperm miRNAs altered by dietary intervention. The final search was completed on March 4, 2025.

### 2.2 Data Extraction and Ingenuity Pathways Analysis

The International Mouse Phenotyping Consortium (IMPC) is a global research initiative aimed at determining the function of every protein‐coding gene in the mouse genome by systematically knocking out each gene and analysing the resulting physiological effects [37, 38]. To support broad usability, the IMPC has created a publicly accessible database of gene functions (https://www.mousephenotype.org/). This resource allows researchers to gain fundamental biological insights, identify disease‐relevant genes, and develop novel therapeutic targets for conditions such as diabetes and rare diseases. Databases such as IMPC also enable large‐scale meta‐analyses, revealing insights that individual studies may miss.

Leveraging this resource, we focused on three broad phenotype categories: abnormal glucose levels, abnormal insulin levels, and body composition (Figure 2B). Each category includes multiple subtypes. In total, nine phenotypes were selected: abnormal glucose levels include *abnormal glucose tolerance, impaired glucose tolerance* and *increased circulating glucose level*; abnormal insulin levels include *decreased circulating insulin level* and *increased circulating insulin level*; body composition encompasses *abnormal lean body mass*, *decreased lean body mass*, *increased bodyweight* and *increased total body fat amount*. This selection (Table 1) resulted in a list of 889 significant candidate genes (Table S1), with body composition accounting for the largest share (540 genes). All downstream analyses were based on this phenotype selection and refer to these categories accordingly.

**TABLE 1:** IMPC mouse phenotypes.

Insulin		Glucose		Body composition	
Phenotype	Number of associated genes	Phenotype	Number of associated genes	Phenotype	Number of associated genes
Increased circulating insulin level	66	Impaired glucose tolerance	199	Increased body weight	16
Decreased circulating insulin level	2	Abnormal glucose tolerance	334	Increased total body fat amount	366
		Increased circulating glucose level	339	Abnormal lean bodymass	674
				Decreased lean body mass	309

We then compared these candidate genes with predicted target genes of sperm miRNAs identified from the literature (Figure 2C). Overlaps were assessed using Venny 2.1 [39]. Inclusion criteria required studies that involved high‐caloric diet interventions and reported altered sperm miRNAs in response to diet [11, 24, 25, 26, 28]. Additionally, we incorporated a dataset of miRNAs dysregulated by acute dietary intervention [19].

Predicted target genes of sperm miRNAs were extracted using miRDB [40, 41]. After curating the target gene lists, we further examined two distinct intervention timeframes: acute HFD feeding (2 weeks) and chronic HFD feeding (7 weeks or more). We compared genes driving metabolic phenotypes with predicted targets from both intervention models (Figure 2A, Table S2).

To gain deeper insight into how miRNA‐regulated genes are linked to metabolic phenotypes, we used Ingenuity Pathway Analysis (IPA) for in silico interrogation of genes regulated by acute (11,272 genes) and chronic (6528 genes) HFD. These analyses revealed six overarching biological categories: *Metabolic and Nutrient Sensing Pathways, Hormone and Endocrine Signalling, Immune Reaction, Neurotransmission and Neuromodulation,* and *Reproduction*.

A limitation of the study is that our analyses are restricted to pre‐clinical mouse models; nevertheless, we have incorporated an overlap with the human genome to enable translational insights, and to prioritise candidate miRNA‐gene interactions for future experimental validation.

### 2.3 Building the ShinyFatSperm App

Following the design principles established in ShinySperm [42] and ShinySpermKingdom [43], we developed a Shiny web application, ShinyFatSperm, to provide a user‐friendly environment for exploring and interpreting the datasets presented in this review. The app was built using the shiny package (v1.10.0) in R (v4.5.0, released 2025‐04‐11) via RStudio (v2025.05.0 + 496).

Additional R packages supporting the app's functionality and visual presentation include DT, eulerr, ggplot2, openxlsx, plotly, readxl, reshape2, RColorBrewer, and shinydashboard.

## 3 Results

### 3.1 Diet Induced Sperm miRNAs Regulate Key Phenotypic Genes

**FIGURE 3:**
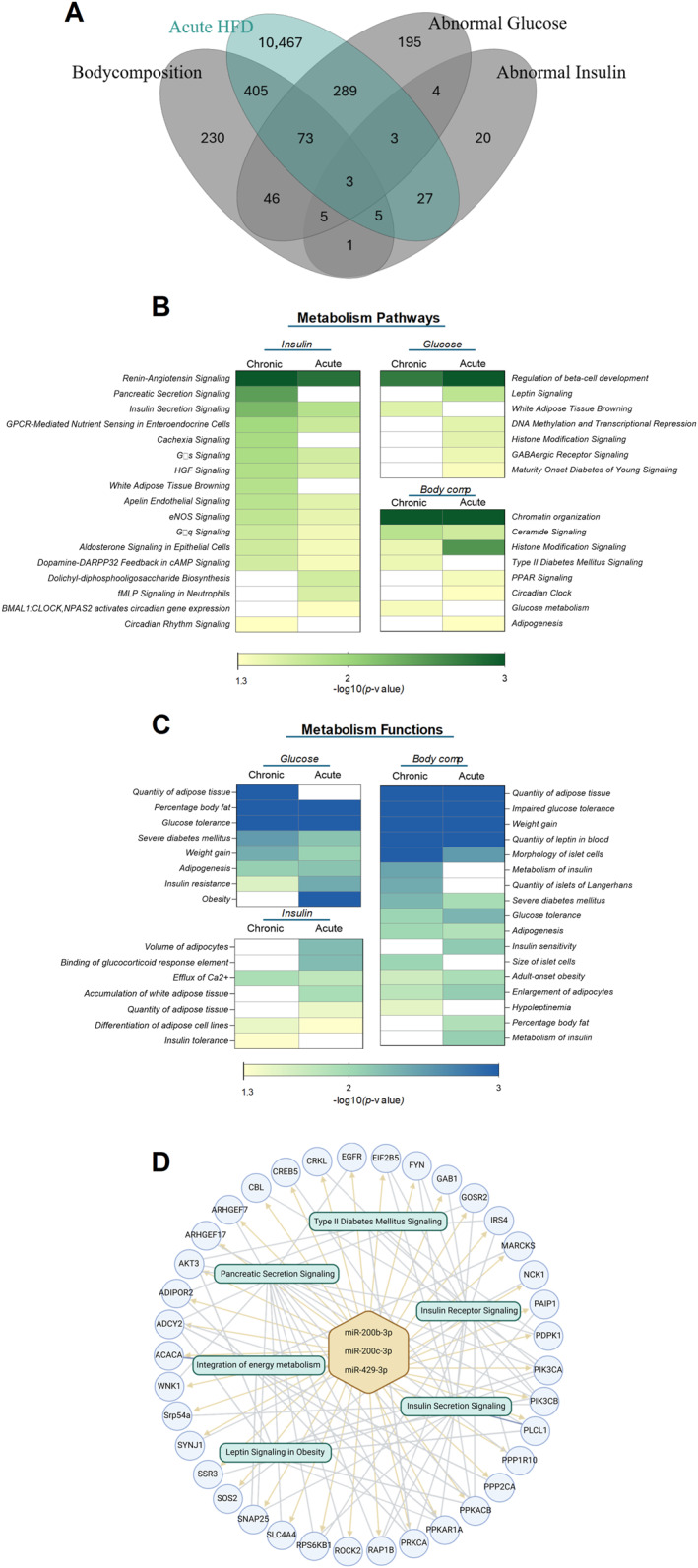
*In‐silico* analysis of acute high fat diet‐regulated sperm miRNAs (phenotype linked) downstream pathways and functions. (A) The VENNY diagram shows the overlap of the phenotype associated genes with miRNA target genes of acute dietary HFD exposure. (B) Ingenuity Pathway Analysis reveals significant different metabolic pathways depending on phenotype and diet exposure. (C) Ingenuity Pathway Analysis reveals significant different metabolic functions depending on phenotype and diet exposure. (D) Example of an integrative network to illustrate how miRNAs dysregulated by HFD may influence pathways and gene expressions.

To explore how diet‐altered sperm miRNAs may be linked to metabolic phenotypes, we intersected predicted gene targets from published miRNA datasets with genes associated with metabolic phenotypes in mice, as annotated by the International Mouse Phenotyping Consortium (IMPC) [37, 38]. In total, 144 unique miRNAs were identified as altered by acute or chronic high‐fat diet (HFD) in sperm based on five published studies [11, 19, 24, 25, 26]. Using miRDB [40, 41], we compiled lists of predicted gene targets for these miRNAs, yielding 11,272 and 6528 target genes for the acute and chronic HFD interventions, respectively (Figure 3A, Table S2).

By overlapping the predicted target genes of sperm miRNAs with our list of 889 significant candidate genes associated with a phenotype, we revealed 805 and 546 genes associated with a metabolic phenotype, and acute or chronic HFD, respectively (Figure 3A, Figure S1).

### 3.2 Case Study: Mrap2 as a Central Node Linking Diet, miRNAs, and Phenotype

Among the three genes shared between acute HFD and obesity‐/diabetes‐associated phenotypes (Figure 3A), Melanocortin‐2 receptor accessory protein 2 (*Mrap2*), Probable E3 ubiquitin‐protein ligase HERC1(*Herc1*), and Trafficking protein particle complex subunit 9 (*Trappc9*), *Mrap2* stands out due to its strong functional link to energy homoeostasis [44]. Loss‐of‐function mutations in *Mrap2* cause early‐onset obesity in both humans and mice [45]. *Mrap2*‐deficient mice exhibit profound obesity through interaction with the melanocortin‐4 receptor (*Mc4r*) in the hypothalamus [46]. Human genetic studies have further identified rare deleterious variants of Mrap2 in individuals with monogenic obesity [47].

In our analysis, *Mrap2* was predicted as a target of miRNA200b‐3p and 200c‐3p as well as 429‐3p (Table S2) following high‐fat diet exposure. Moreover, *Mrap2* in pancreatic δ‐cells is critical for ghrelin‐induced insulin inhibition [48] and plays a key role in regulating ghrelin receptor signalling and hunger perception [49]. Given *Mrap2*'s critical role in MC4R signalling, leptin responsiveness, and body weight [45], this finding highlights a plausible mechanistic link whereby paternal diet could modulate offspring appetite and energy balance via sperm miRNAs.

### 3.3 Overlapping and Distinct miRNA–Gene Networks in Acute Versus Chronic HFD

We next examined the overlap of individual miRNAs and their predicted target genes between acute and chronic HFD exposure (Figure 3B,C). The overlap of miRNAs between both conditions was modest, suggesting that time‐dependent differences exist in the small RNA cargo of sperm. Similarly, most target genes were specific to either acute or chronic HFD. Only 144 genes were shared between the two models. This pattern reflects the dynamic adaptation of the epididymal environment.

Diving deeper into the analysis, it is also possible to look at overlaps of specific phenotypes, for example glucose phenotype, with the miRNA target genes. Interestingly, amongst the shared genes of the glucose phenotype and acute, as well as chronic HFD feeding is exemplary peroxisome proliferator‐activated receptor gamma (*Pparγ*) (Table S2). *Pparγ* is a key regulator of adipogenesis and insulin sensitivity [50], exerting its effects primarily through adipose tissue by modulating genes involved in lipid metabolism and insulin signalling. Its activation improves whole‐body insulin sensitivity and glucose homoeostasis, partly through enhanced fatty acid storage and anti‐inflammatory cytokine regulation. Genetic and post‐transcriptional variations in *Pparγ* influence its activity and are linked to adipose dysfunction, insulin resistance, and variable responses to *Pparγ* ‐targeted therapies in type 2 diabetes [50, 51]. This result provides both a further validation of the approach and an intriguing finding, as it reveals an overlap between acute and chronic interventions despite their impact on distinct pathways and functions (Figure 3B,C). In general, the pattern likely reflects the dynamic adaptation of the epididymal environment and supports the hypothesis that acute dietary exposures may trigger more sharply defined and functionally specific epigenetic responses, compared to the broader or compensatory shifts seen with prolonged nutritional stress.

### 3.4 Pathway Analysis Reveals Functional Clustering of Candidate Genes

Using Ingenuity Pathway Analysis (IPA), we identified major biological categories enriched among miRNA target genes derived from acute or chronic HFD exposure. Within *Metabolic and Nutrient Sensing Pathways*, we observed pathways associated with abnormal insulin phenotypes that were more significantly enriched in pathways such as *pancreatic* and *insulin secretion signalling* during chronic HFD exposure compared with acute feeding (*p*‐value of 0.004 and 0.006 vs. 0.015 respectively) (Figure 3B, Table S3). Notably, the heat map also reveals substantial overlaps between the two feeding periods, suggesting shared underlying mechanisms. For example, both chronic and acute interventions affected genes which are linked to the abnormal insulin phenotype and regulate *beta‐cell development*. However, certain pathways are exclusively regulated during either acute or chronic HFD feeding. Notably, leptin signalling was exclusively regulated by acute HFD in our analyses; this may be attributable to the development of leptin resistance that occurs during prolonged periods of high‐fat diet feeding [52]. Similar observations were made regarding metabolic functions (Figure 3C). Overlaps between acute and chronic HFD feeding were observed in the regulation of key processes such as weight gain, percentage body fat and impaired glucose tolerance. In contrast, functions such as islet cell size and insulin tolerance appear to be specifically regulated only after chronic HFD feeding. To support these observations, we include an illustrative integrative network demonstrating how miRNAs dysregulated by acute HFD exposure are predicted to regulate gene networks converging on obesity‐related pathways such as *leptin signalling in obesity* (Figure 3D).

The remaining five broad biological categories are also further subdivided into pathways and functions associated with the respective phenotypes (Figures [Link], [Link], Table S4). To facilitate researchers to explore these in silico analysis and generate their own hypotheses, we developed the ShinyFatSperm App.

### 3.5 The ShinyFatSperm App Enables Interactive Exploration of miRNA–Phenotype Links

To facilitate transparent and user‐friendly access to these findings, we developed the *ShinyFatSperm* App (https://reproproteomics.shinyapps.io/ShinyFatSperm/). This tool allows users to explore which miRNAs are altered by HFD, their predicted gene targets, and how these genes relate to metabolic phenotypes annotated by the IMPC. Users can query overlaps between acute and chronic HFD models, visualise Venn diagrams, and generate pathway enrichment plots. This application enables hypothesis generation and fosters reproducibility by directly connecting experimental miRNA data with phenotypic gene annotations in an interactive format.

## 4 Discussion

Obesity and diabetes are global health crises whose rising prevalence has prompted intense investigation into their biological origins [1, 2, 3]. While traditional research has emphasised genetic predisposition and environmental risk factors, growing evidence highlights the importance of epigenetic inheritance, particularly through paternal sperm, in shaping offspring metabolic health [11, 19, 28]. Sperm contributes not only DNA but also small non‐coding RNAs, such as microRNAs (miRNAs) that can modulate gene expression and thereby influence intergenerational disease risk. This emerging paradigm calls for an integrated understanding of epigenetic mechanisms in metabolic disease aetiology.

One striking example illustrating the complexity of intergenerational transmission is the miRNA‐mediated regulation of *Mrap2*, a gene strongly implicated in energy homoeostasis. *Mrap2* loss‐of‐function mutations lead to early‐onset obesity in mice and humans [45]. In our analysis, *Mrap2* was identified as a predicted target of miRNA200b‐3p and 200c‐3p, as well as 429‐3p. This suggests a plausible pathway through which paternal diet could influence hypothalamic energy regulation in the offspring, underlining the biological relevance of sperm miRNA–gene interactions [46]. Moreover, diet‐induced leptin resistance, a hallmark of chronic HFD feeding [52], may further modulate this axis, strengthening the rationale for integrative approaches like ours that connect sperm miRNAs to phenotype‐associated genes.

The duration of dietary exposure appears to be critical in determining the nature of epigenetic responses. However, the underlying mechanisms driving the differential effects of dietary interventions, such as chronic versus acute HFD exposure, remain not completely understood. Nevertheless, several hypotheses have been proposed, including distinct epigenetic responses to varying durations of dietary intake. Acute HFD exposure may cause temporary changes in gene expression and epigenetic modifications that the body can resolve with time and diet change [19]. In contrast, chronic exposure likely causes persistent, potentially maladaptive epigenetic alterations, which may exacerbate obesity and diabetes. However, both acute and chronic HFD exposure, if not resolved with a diet change, can affect sperm miRNAs, altering the epigenetic programming of the offspring [11, 19, 24, 25, 26]. Nevertheless, chronic diet‐induced changes in sperm epigenetics may be more detrimental in passing on obesity and diabetes compared with acute changes.

The epididymis plays a central role in this context, mediating the final steps of sperm maturation and sncRNA acquisition [18]. Given that the post‐testicular phase has already been detailed in the Introduction [13, 14, 17, 18, 19, 20, 21], we refer the reader to that section for mechanistic insights into soma‐to‐germ communication and the role of epididymosomes. Here, we emphasise that the dynamic nature of the epididymal microenvironment offers a sensitive window through which environmental factors such as diet may shape sperm RNA content and ultimately influence offspring phenotypes.

Despite the growing body of evidence supporting the role of sperm epigenetics in the inheritance of metabolic diseases, a critical link remains elusive: how do altered sperm miRNAs influence gene expression in offspring? A potential mechanism could be that sperm miRNAs may regulate critical metabolic genes post‐fertilisation. If these miRNAs are passed on to the next generation, they could adapt important metabolic genes to an energy rich environment [53], and prime offspring metabolism for energy rich environments. These findings point to a possible epigenetic mechanism where the father's diet affects metabolic traits in future generations and propose a mechanism for epigenetic inheritance of obesity and diabetes risk.

To address this gap, we systematically linked diet‐regulated sperm miRNAs to phenotype‐associated gene sets using publicly available datasets [11, 19, 24, 25, 26] and resources, including miRDB and the International Mouse Phenotyping Consortium (IMPC) [37, 38]. Our analyses revealed distinct miRNA–gene networks in acute versus chronic HFD models, including shared nodes in pathways related to insulin signalling, lipid metabolism, and β‐cell function. By offering the ShinyFatSperm App as an interactive exploration tool, we aim to make these findings accessible and stimulate further research into paternal epigenetic inheritance.

While miRNAs are the most studied class of sncRNAs, other species such as mitochondrial RNAs and piwi‐interacting RNAs (piRNAs) are likely to play complementary roles [19]. However, the lack of predictive bioinformatic frameworks for these molecules limits our current understanding. Future studies should prioritise the development of dedicated tools and functional assays, such as sRNA tools [54], to dissect their contributions.

Taken together, our findings reinforce the concept that paternal dietary exposures can influence offspring health through epididymal‐ and sperm‐borne miRNAs, and related epigenetic mechanisms. This work provides a roadmap for hypothesis‐driven investigation into the intergenerational inheritance of metabolic diseases and highlights the urgent need for translational strategies to interrupt this cycle. For future investigations, these findings have potential clinical implications beyond improving our understanding of obesity and diabetes mechanisms. Diet‐responsive sperm miRNAs may represent accessible epigenetic biomarkers of paternal metabolic health and intergenerational disease risk. In clinical assisted reproduction settings, such signatures could contribute to risk stratification or counselling strategies by identifying epigenetically compromised sperm populations; future work may lead to sperm selection based upon epigenetic cargo. More broadly, sperm miRNA profiling could support preconception screening and guide lifestyle, or dietary interventions, aimed at reducing the transmission of metabolic dysfunction to subsequent generations.

## Author Contributions

**Katharina Laurent:** conceptualization, methodology, investigation, writing – original draft, writing – review and editing, visualization. **Raffaele Teperino:** resources, writing – review and editing, funding acquisition. **Martin Hrabě de Angelis:** resources, writing – review and editing, funding acquisition. **David A. Skerrett‐Byrne:** conceptualization, resources, methodology, investigation, software, writing – original draft, writing – review and editing, visualization, funding acquisition, supervision. **Johannes Beckers:** conceptualization, resources, investigation, writing – original draft, writing – review and editing, visualization, funding acquisition, supervision.

## Conflicts of Interest

The authors declare no conflicts of interest.

## Supporting information


**Figure S1:** Comparative analysis of high fat diet (HFD) induced sperm miRNAs and metabolic phenotypes. Venn diagrams illustrate the overlap between metabolic phenotype‐associated genes and predicted target genes of sperm miRNAs following chronic HFD exposure only, and the combination of chronic and acute dietary HFD exposure.

**Figure S2:** Pathway‐level enrichment of phenotype‐linked sperm miRNAs target genes regulated by high‐fat diet (HFD). Heatmaps display enriched biological pathways associated with sperm miRNAs altered by acute and chronic HFD, highlighting pathways related to immune regulation, hormonal signalling, and neurological processes.

**Figure S3:** Downstream molecular function analysis of phenotype‐linked sperm miRNAs target genes regulated by high‐fat diet (HFD). Heatmaps compare downstream molecular functions associated with sperm miRNAs altered by acute and chronic HFD, with enrichment in immune, hormonal, and reproductive functional categories.

**Table S1:** IMPC genes of metabolic phenotypes.

**Table S2:** Predicted target genes of miRNAs identified in epididymal sperm epigenome studies.

**Table S3:** Ingenuity Pathway Analysis—pathways outputs.

**Table S4:** Ingenuity Pathway Analysis—disease & function outputs.

## Data Availability

The data that support the findings of this study are openly available in ShinyFatSperm at https://reproproteomics.shinyapps.io/ShinyFatSperm/.
