# Supplementary figures and images for: Epigenetic Legacy: The Role of Sperm miRNAs in the Paternal Inheritance of Diabetes and Obesity Development
Source: Diabetes Metab Res Rev. 2026 Mar 23;42(3):e70157. doi: 10.1002/dmrr.70157 (PMC13006935; doi:10.1002/dmrr.70157)

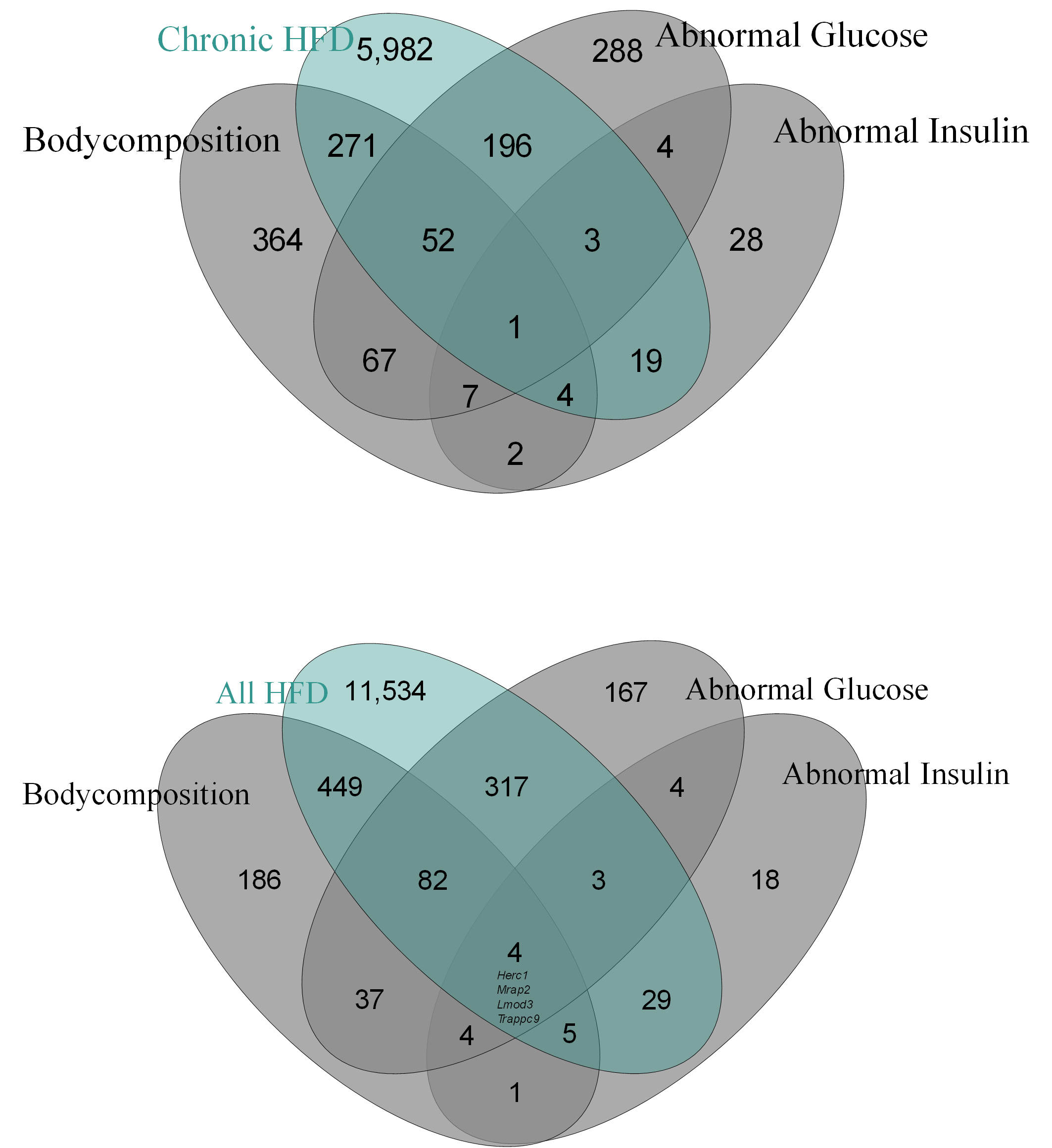

Supplement: Supplementary file 1 — Figure S1: Comparative analysis of high fat diet (HFD) induced sperm miRNAs and metabolic phenotypes. Venn diagrams illustrate the overlap between metabolic phenotype‐associated genes and predicted target genes of sperm miRNAs following chronic HFD exposure only, and the combination of chronic and acute dietary HFD exposure. [file DMRR-42-e70157-s004.tif]

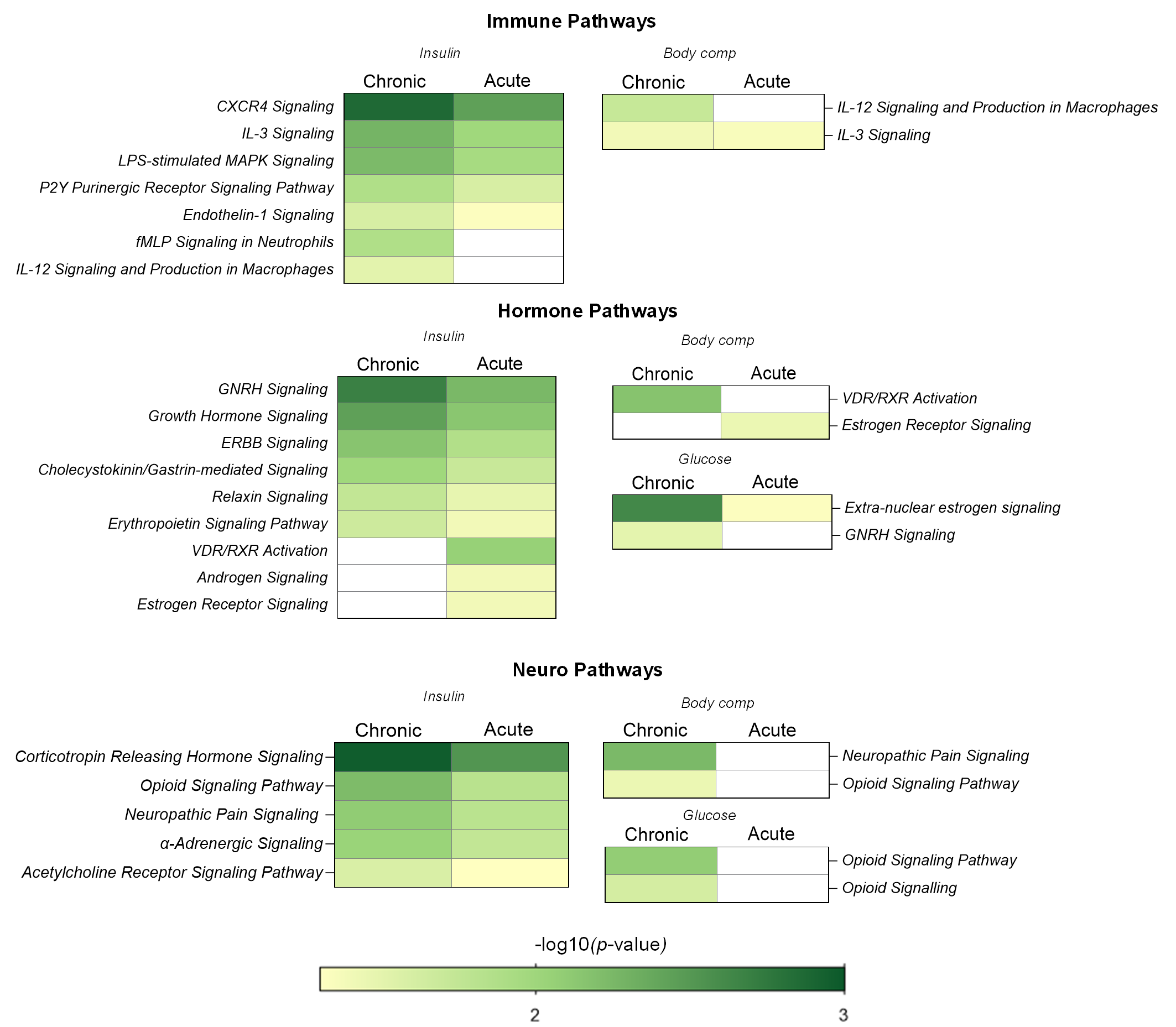

Supplement: Supplementary file 2 — Figure S2: Pathway‐level enrichment of phenotype‐linked sperm miRNAs target genes regulated by high‐fat diet (HFD). Heatmaps display enriched biological pathways associated with sperm miRNAs altered by acute and chronic HFD, highlighting pathways related to immune regulation, hormonal signalling, and neurological processes. [file DMRR-42-e70157-s003.tif]

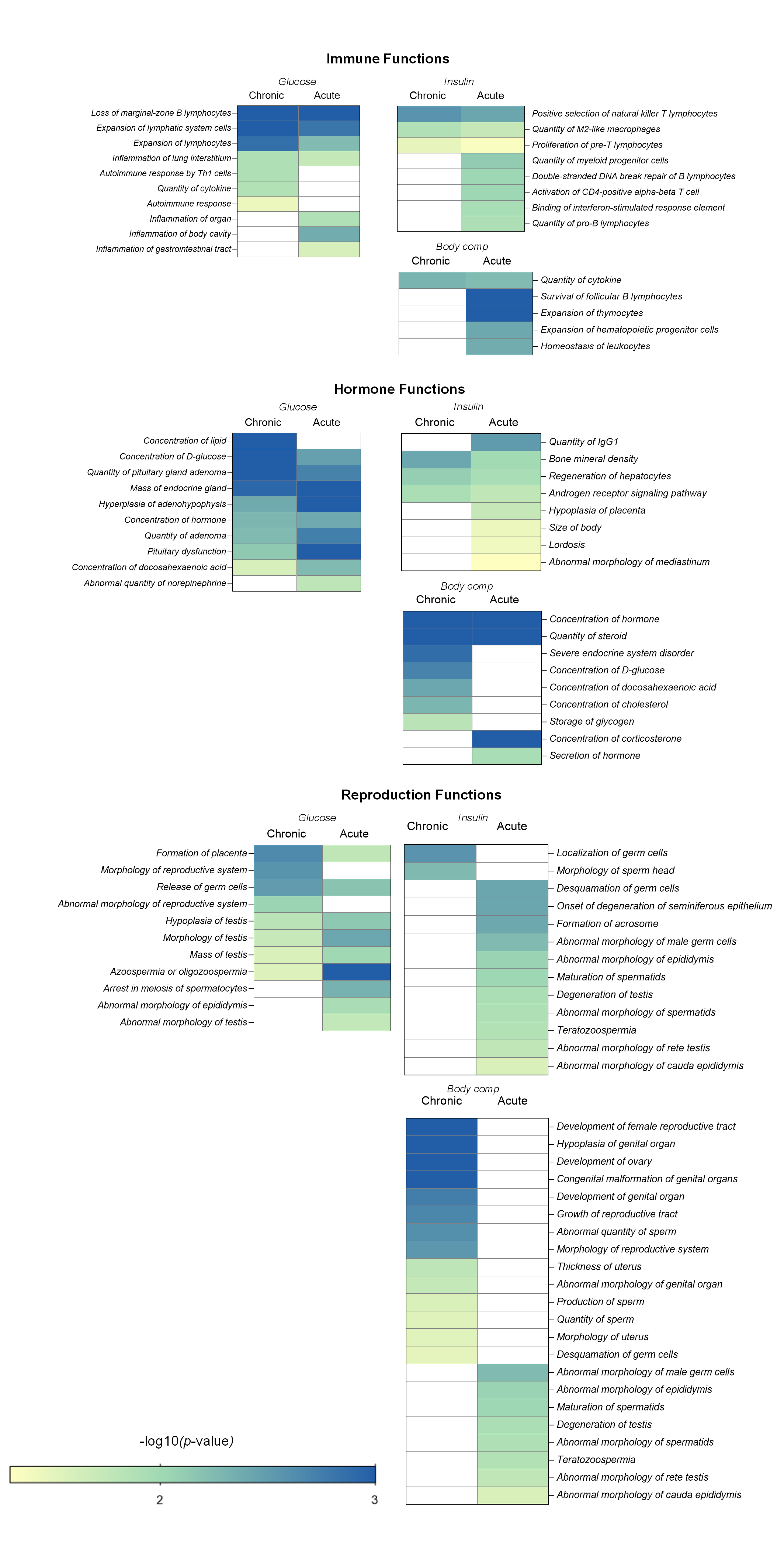

Supplement: Supplementary file 3 — Figure S3: Downstream molecular function analysis of phenotype‐linked sperm miRNAs target genes regulated by high‐fat diet (HFD). Heatmaps compare downstream molecular functions associated with sperm miRNAs altered by acute and chronic HFD, with enrichment in immune, hormonal, and reproductive functional categories. [file DMRR-42-e70157-s007.tif]
